# A new method for measuring the size of nematodes using image processing

**DOI:** 10.1093/biomethods/bpz020

**Published:** 2019-12-27

**Authors:** David G H Andrews

**Affiliations:** School of Engineering, Technology and Design, Canterbury Christ Church University, North Holmes Road, Canterbury CT1 1QU, UK

**Keywords:** nematode, size, image processing

## Abstract

Many studies have been made on nematodes, especially *Caenorhabditis Elegans*, which are used as a model organism. In many studies, the size of the nematode is important. This article describes a method of measuring the length, volume and surface area of nematodes from photographs. The method uses the imaging software ImageJ, which is in the public domain. Two macros are described. The first converts the images into binary form, and the second uses several built-in functions to measure the length of the worm and its diameter along its length. If it is assumed that the worm has a circular cross-section, then the volume and surface area of the nematode can be calculated. This is a cheap and easy technique.

## Introduction

The phylum Nematoda is very diverse, and nematodes are widely studied. The nematode *Caenorhabditis elegans* has been used as a model for ageing [1] for population studies [2] and for genomics modelling [3, 4]. *Caenorhabditis* *elegans* have a quick life cycle (∼2.5 days) and a short life span (∼2 weeks), and they are fairly small (an adult worm has a diameter of about 50 μm and about 1 mm long.) In many studies, the length of the worm has been measured. Since living worms are very seldom straight, this is not always straightforward. This measurement was performed by drawing a series of segmented lines along the worm using image processing software such as ImageJ [5] and adding the lengths [6, 7]. Some studies use the area of the nematode, measured using image processing, as a measure of the size of the worm [8]. Another technique is to measure them when straightened after death [9, 10] or chemically treated [11]. In several studies, it is important to be able to measure the volume of the worms [12, 13]. In the past, this was usually done by measuring the length and diameter of the worm and assuming they were cylindrical [14–16]. This technique will tend to overestimate the volume. Recently, there have been developments using microfluidic chips to measure the size of the worms, for instance, Hu *et al.* [17] described a microchip for sorting *C. elegans* by size. There have also been some specialized image processing techniques, such as that described in [18].

In this article, an image processing technique for measuring the volume of *C. elegans* using open-source image processing software, Fiji [19], which is a version of ImageJ, is described. Photographs of the individual worms were taken using a microscope, and the photographs cropped to show individual worms. Since the processing involved can be written as a macro, large numbers of images can be processed in a short time. The advantage of this system is that it is free.

## Material and Methods

As stated in the introduction section, this method only works for one worm per image, so it is necessary to crop each image so that one worm is present, and other objects, such as eggs, are excluded. Also, the images should be spatially calibrated, so the output is in the units of length rather than pixels.

**Figure 1:**
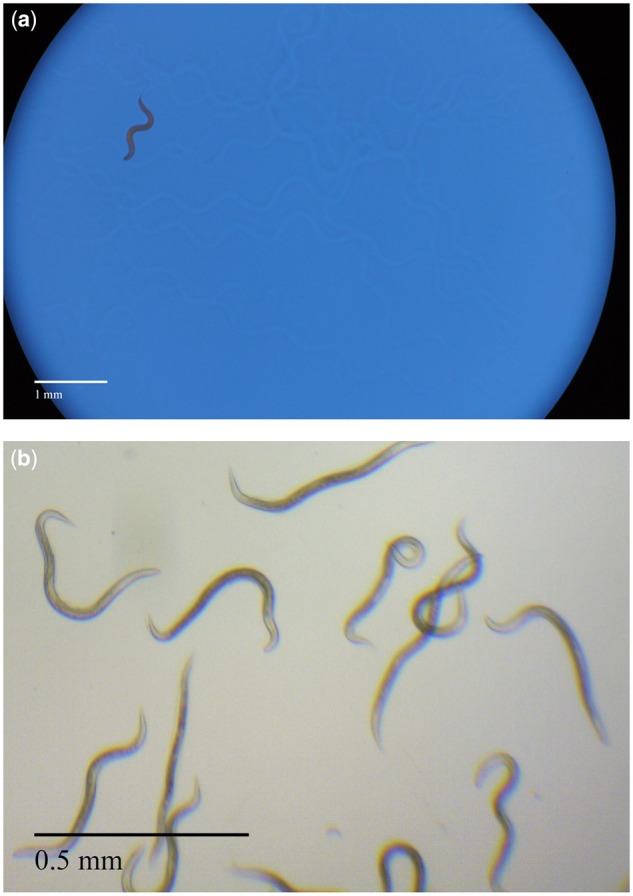
(**a**) *Caenorhabditis elegans* on agar photographed with a Nikon camera through a Leica microscope. (**b**) Nematodes photographed in water using a Leica microscope with a built-in camera.

The first step is to make the image binary, with the worm black and the background white. How this is done depends on the initial image. Figure 1a shows a *C. elegans* photographed on agar on a Petri dish through a microscope. Blue light was used to enhance the contrast between the worm and the background. Figure 1b shows the nematodes photographed in a water droplet on a slide. It is important to achieve the greatest contrast in the initial image.

**Figure 2:**
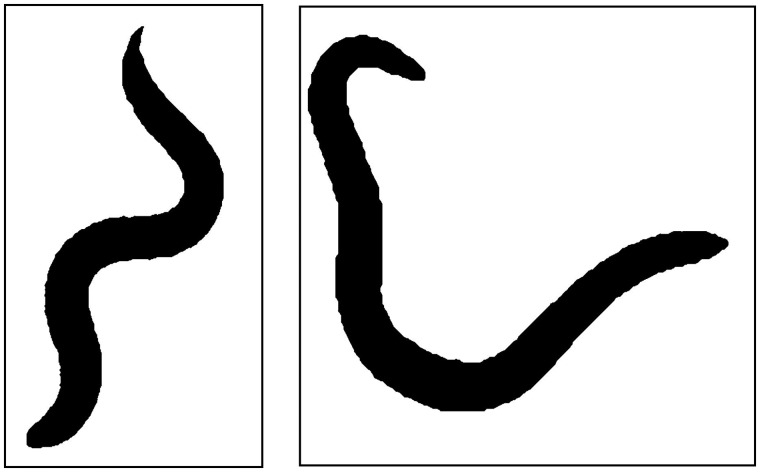
Binary images of nematodes are shown in Figure 1.

After selecting a small area around the worm, the image is split into the red, green and blue components, and the one with the greatest contrast is selected. The ‘enhanced contrast’ tool is then applied, followed by the ‘binarize’ tool. In some photographs, after binarizing, the ‘fill-holes’ tool is needed. If the edges of the image are not sharp, then the image can be dilated then eroded, and if there are other small artefacts in the image, such as eggs, then these can be eliminated by eroding followed by dilating. The binary images for the above two pictures are shown in Fig. 2. The final part of this initial processing is to expand the canvas and rotate the image until its longest dimension is horizontal, and the image saved into a separate folder. If many worms are to be analysed, then this initial processing can be automated using a macro. An example of such a macro (binary.ijm) can be found at https://github.com/Hornerman/nematode, but it will need to be adapted depending on the quality and colours in the original images. After downloading the macro, it should be run from Fiji. This macro makes use of several built-in functions available in Fiji, such as ‘make binary’. You will be asked to select the folder, which should have a subfolder called ‘photos’ where the images of the worms should be placed with no other files. The binary images will be placed in a subfolder called ‘binary’. Some initial changes to the image may be needed, such as contrast, to produce a good binary image.

**Figure 3:**
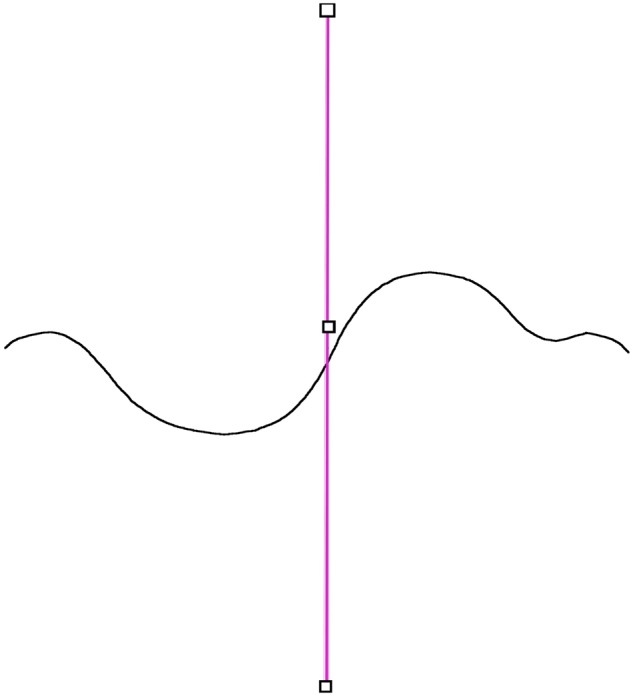
Line through the centre of ‘spine’ line.

**Figure 4:**
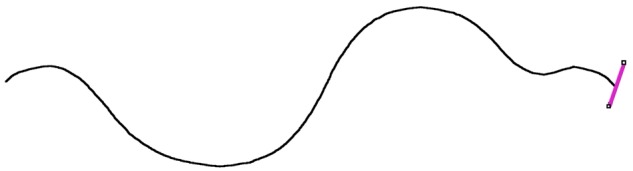
‘Spine’ line of the worm, with the line to find its end.

The Fiji macro (worm.ijm which is also at https://github.com/Hornerman/nematode) is designed to find the length, volume and surface area of the worm uses several built-in functions provided in Fiji. The worm is turned into a line using the built-in ‘2 D/3D Skeletonize’ tool which erodes the image to a line one pixel wide. This line will be referred to as the ‘spine line’. A function within the programme called ‘beginning’ then finds the end of the spine line. A vertical profile line is drawn through the centre of the image (Fig. 3) and the intensity profile along it is measured. The maximum on the profile gives a point on the spine line. If the worm is curved, then there may be more than one point, so the first point is chosen. A second vertical profile line is drawn a few pixels to the right of the first line, and a second point on the spine line is found. This allows the position and slope of the spine line to be determined. After this, a short profile line (about 40 pixels long) is drawn perpendicular to the spine line. It is moved along the line, calculating the position and the slope at each point to keep the line segment perpendicular. This is continued to the end of the spine line. This is shown in Fig. 4. A second function called ‘points’ moves the short profile lined through the worm to the other end. This process gives in the coordinates and slope of the spine line along its length, from which the length of the line can be found. A limitation of the method is if part of the spine line gets closer than about 20 pixels or overlaps another part, it will fail.

**Figure 5:**
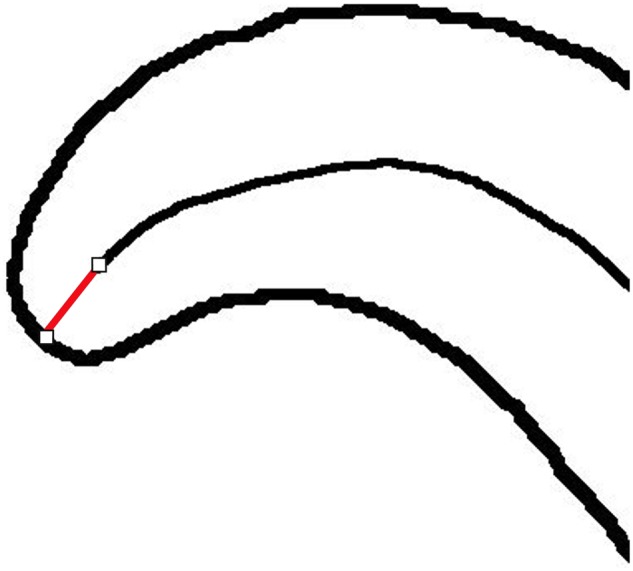
Extending ‘spine’ line to the end of the worm.

The spine line produced by the 2D/3D Skeletonize tool is slightly shorter than the worm, due to the eroding action of the tool. A function ‘ends’ extends the spine line to the ends of the worm. The original binary image is converted to a one-pixel wide outline using the built-in tool ‘Outline’. The resulting image is called ‘edge.tif’. The slope of the beginning and end of the spine line is measured, and a profile line drawn at this slope from the end of the thin spine line to the outline of the complete worm (Fig. 5). This allows the spine line to be extended to the complete length of the worm. The total length of the worm is given by adding the extra lengths to the original length of the spine line.

**Figure 6:**
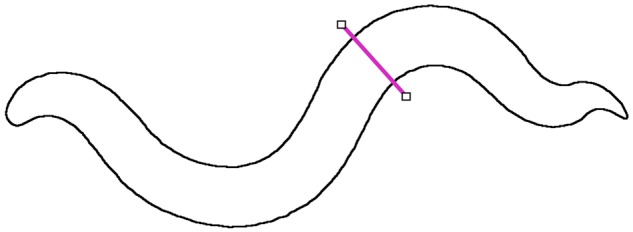
Find the width of the worm.

The function ‘radii’ finds the positions of the edge of the outline from the ‘edge.tif’ image. A slightly longer line is moved through the outline of the worm drawn normal to the central line. This is shown in Fig. 6. This gives the diameter of the worm along its length.

**Figure 7:**
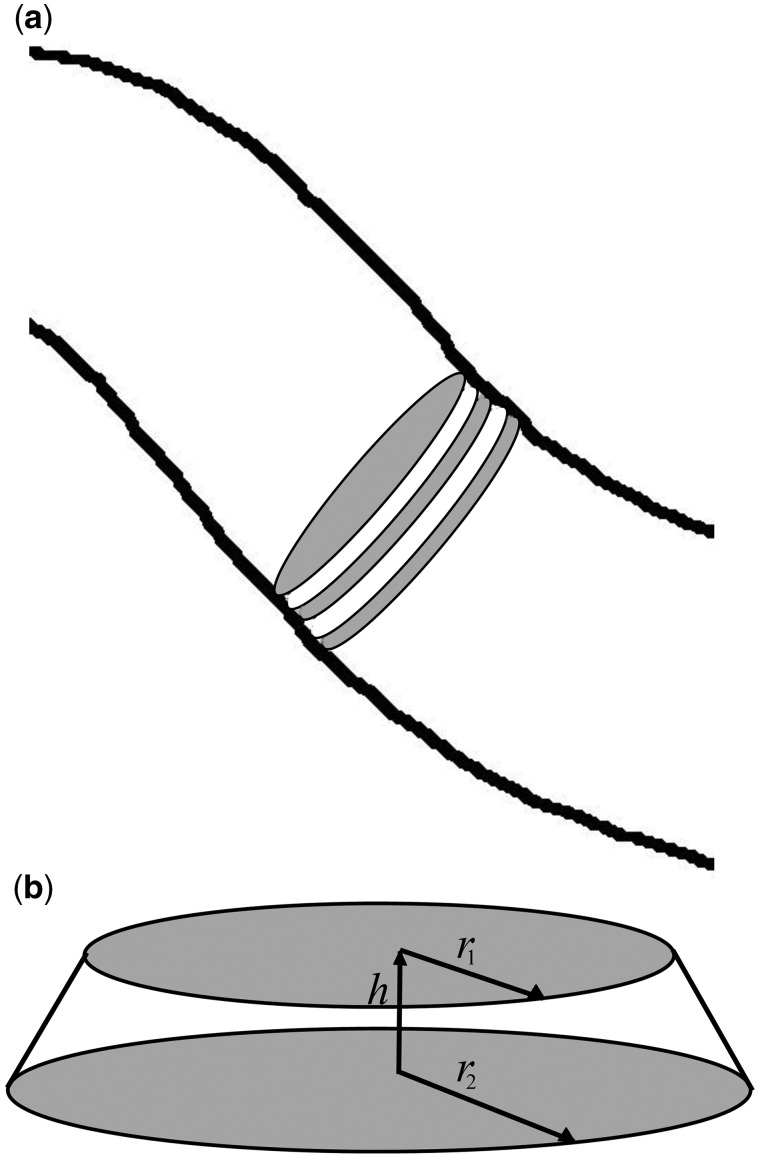
(**a**) Worm divided into truncated cones. (**b**) Truncated cone.

To find the volume of the worm, assume it is divided up into a series of truncated cone sections, as shown in Fig. 7a. The volume of each section is calculated, assuming it is a circular truncated cone, height h and radius at either end $r_{1}$ and $r_{2}$ (Fig. 7b) using the following equation:

$$V=\frac{1}{3}\pi \left(r_{1}^{2}+r_{1}r_{2}+r_{2}^{2}\right)h$$

The surface area of the worm can be found by summing the lateral surface area of the truncated cones:

$$S=\pi \left(r_{1}+r_{2}\right)\sqrt{{\left(r_{1}-r_{2}\right)}^{2}+h^{2}}$$

**Figure 8:**
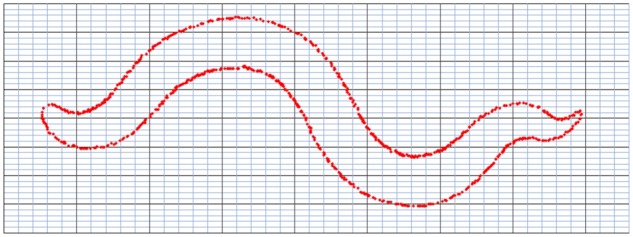
Worms outline are shown in Excel.

**Figure 9:**
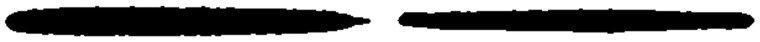
Straightened silhouettes of the worms in Figure 1.

The outputs from the programme are a text file from for each worm, labelled output*filename*.txt and a text file labelled output.txt for the complete set of worms. output*filename*.txt lists the positions of the edges of the worm, and output.txt lists the length, volume and surface area of each worm. These files can be used in another programme such as Excel (Fig. 8). Also, a straightened silhouette of the worm can be produced (Fig. 9).

With minor changes to the programme, the curvature of the worm k=dϕ/ds could be calculated, where dϕ is the change in angle for each distance ds along the worm from the spine line (Fig. 4). This quantity is important in some studies, for example, Padmanabhan *et al*. [20].

## Results

The software has been tested on several sets of worm images. The results are exported as text files. An example of some of the measurements on for a single worm is given in Table 1, showing the position along the worm and width at that point. Table 2 is an example of measurements on a set of worms giving the length, volume and surface area.

**Table 1:** Sample of data from a single worm

Position	Width
233.257	347.951
237.658	350.324
241.614	349.73
245.718	350.126
252.002	351.974
254.144	352.863
258.262	354.134
262.457	354.407
266.258	354.72
270.389	354.999
274.816	355.262
278.406	355.599
283.24	354.806
286.984	355.132
290.644	355.457
294.84	354.738
298.9	356.071
301.897	356.352
305.364	357.607
307.613	357.61
310.416	358.508

**Table 2:** Sample of data from a set of worms

File	Length (mm)	Volume (mm^3^)	Surface area (mm^2^)
1.tif	1.247	0.00594	0.2918
10.tif	1.294	0.00598	0.2939
11.tif	1.262	0.00592	0.2934
12.tif	1.28	0.00602	0.2977
13.tif	1.246	0.006	0.2945
14.tif	1.252	0.00594	0.2925
15.tif	1.256	0.00599	0.2928
16.tif	1.25	0.00595	0.2924
17.tif	1.27	0.00596	0.2947
18.tif	1.286	0.00596	0.2951
2.tif	1.254	0.00595	0.2934
3.tif	1.248	0.00593	0.2908
4.tif	1.252	0.00599	0.2931
5.tif	1.215	0.00584	0.2827
6.tif	1.251	0.00593	0.2919
7.tif	1.259	0.00598	0.292
8.tif	1.271	0.00599	0.2957
9.tif	1.268	0.00596	0.2934

When applied to a set of 18 different images of the same worm, the standard deviation (SD) of the lengths was <2.5% of the average length, the SD of the surface areas was <4% of the average surface area and the SD of the volumes was 2% of the average volume.

**Figure 10:**
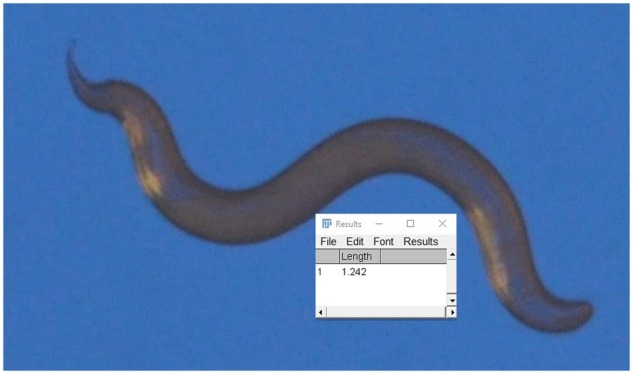
Segment line through the worm.

It is possible to measure the length of the worms manually using an image package such as ImageJ. If the line is drawn along the worm, then the programme gives its length. This is a fairly time-consuming process. In Fig. 10, segmented line through the worm had length 1.24 mm, which was in very good agreement with the length 1.247 mm given by the automated worms.ijm macro. The maximum width of this worm was measured manually to be about 0.1 mm. If the worm was assumed to be cylindrical, then the resulting volume would be 0.0097 mm^3^. This is significantly greater than the value of 0.00594 mm^3^ given by the macro since the macro actually measures the shape of the worm.

## Discussion

The system gives an easy way of comparing the size of worms, though it was not compared to any other measurement system.

The quality of the initial photograph is very important. The greater the initial contrast between the worm and its surroundings, the easier it is to produce a binary image. With good quality images, the size of large numbers of nematodes can be measured very easily and quickly. The main limitation of the technique is that it cannot deal with worms overlapping themselves or other worms.

The resolution of the measurements is dependent on the number of pixels within each worm. The method counts the number of pixels around the perimeter of the worm, so the greater the number of pixels, the greater the resolution.
